# A critical review on the roles of natural products in shaping oral microbiota and preventing chronic diseases

**DOI:** 10.1007/s13659-026-00605-3

**Published:** 2026-03-04

**Authors:** Yogesh Kumar, Baojun Xu

**Affiliations:** 1 Department of Biotechnology, Mehsana Urban Institute of Sciences, Ganpat University, Kherva, Gujarat 384012 India; 2 Food Science and Technology Program, Department of Life Sciences, Beijing Normal-Hong Kong Baptist University, Zhuhai, 519087 Guangdong China

**Keywords:** Oral microbiota, Dysbiosis, Natural products, Chronic diseases, Microbiome modulation, Antimicrobial agents

## Abstract

The oral microbiome plays a central role in maintaining both oral and systemic health, and disruptions in its balance can contribute to a wide range of diseases. This review brings together current evidence on how natural products modulate oral microbial communities, promote microbial equilibrium, and help prevent conditions such as dental caries, periodontitis, and chronic systemic disorders linked to oral dysbiosis. Recent studies highlight that phytochemicals particularly polyphenols, terpenoids, saponins, and alkaloids exert antimicrobial, anti-inflammatory, and antioxidant effects that influence bacterial adhesion, biofilm development, gene expression, and acid production. These compounds not only inhibit key oral pathogens but also support beneficial species, helping to sustain a stable and resilient microbiome. Evidence was gathered from PubMed, Scopus, ScienceDirect, and Google Scholar using relevant keywords and focusing on literature from 2015 to 2025. Insights into microbial diversity, environmental influences, host genetics, and advanced sequencing tools further strengthen understanding of oral microbial dynamics. While natural products show strong potential, challenges related to safety, bioavailability, regulatory clarity, and clinical translation remain to explore. This review outlines current progress and future directions needed to transform natural compounds into effective, evidence-based strategies for improving oral and systemic health through microbiome modulation.

**Graphical Abstract:**

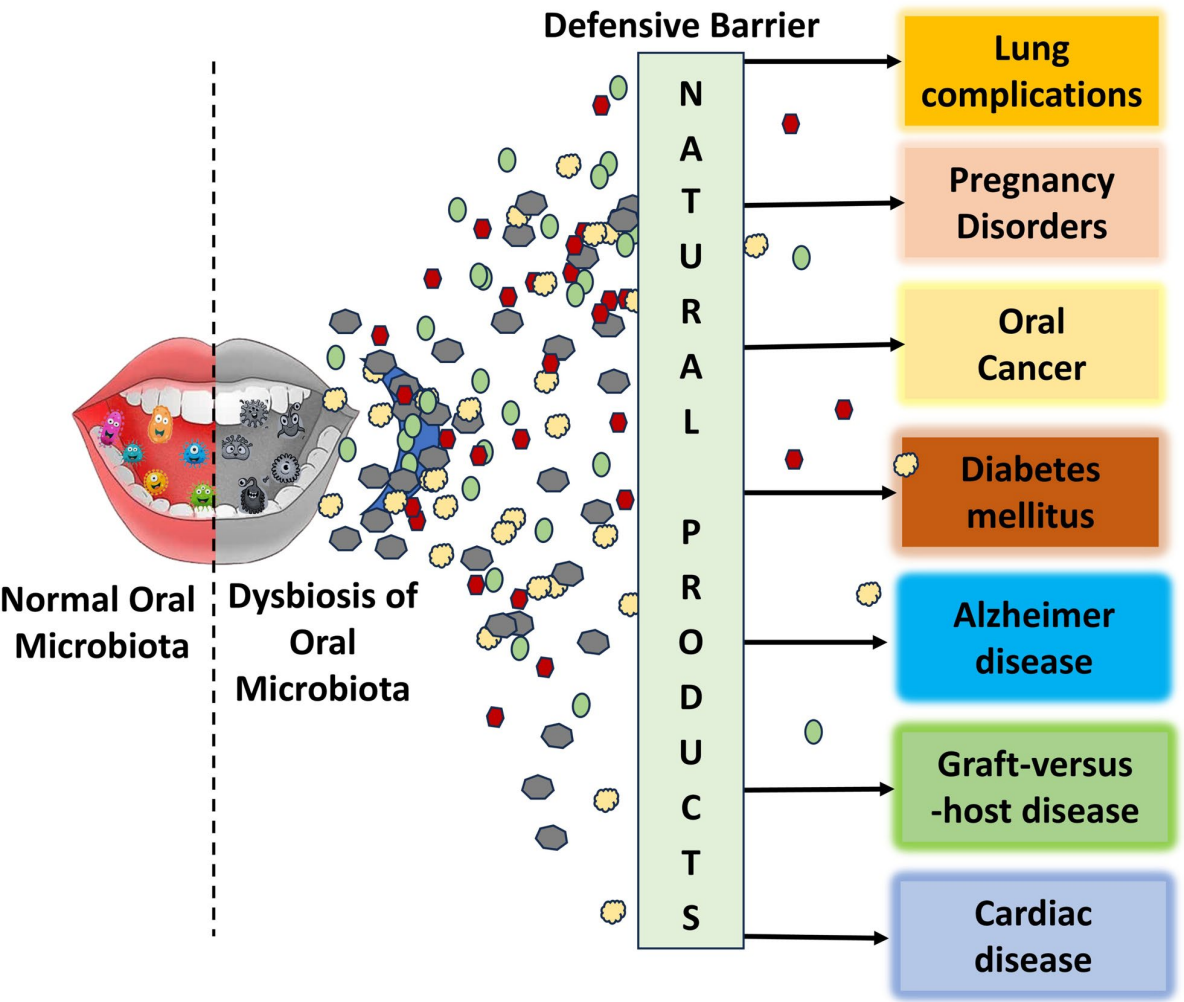

## Introduction

The oral microbiota plays a critical role in maintaining both oral and systemic health. It functions as a dynamic ecological community composed of bacteria, fungi, viruses, and archaea that coexist with the host in a delicate balance. This microbial ecosystem resides in various niches within the oral cavity, including the tongue, teeth, gingival sulcus, and mucosal surfaces, where it contributes to immune modulation, nutrient metabolism, and resistance to pathogenic invasion. The oral cavity, being one of the primary interfaces between the body and the external environment, facilitates constant microbial exchange, making the oral microbiota an important indicator of overall health status [1–3]. Disruption of this microbial balance, known as dysbiosis, has profound implications not only for oral diseases such as dental caries and periodontitis but also for systemic disorders. Inflammatory conditions such as diabetes, rheumatoid arthritis, and systemic lupus erythematosus have demonstrated a direct association with altered oral microbial profiles and increased pathogenicity. For example, increased levels of *Porphyromonas*, *Capnocytophaga*, and *Pseudomonas* have been identified in diabetic patients, while *Prevotella* and *Selenomonas* dominate in individuals with autoimmune conditions. These systemic diseases often exacerbate inflammation in the oral cavity, contributing to periodontal tissue destruction and alveolar bone loss. Furthermore, oral pathogens have been detected in distal organs, suggesting that the oral cavity may serve as a reservoir for systemic infections [4–6].

The influence of the oral microbiota on systemic health is mediated through complex immune interactions. Dysbiotic microbial communities stimulate excessive immune responses that lead to chronic inflammation. Elevated levels of cytokines such as interleukin-17 (IL-17) are observed in both oral and systemic inflammatory diseases, and targeted inhibition of IL-17 has shown potential in reversing oral microbial pathogenicity [4, 6]. Moreover, microorganisms originating from the oral cavity can enter the bloodstream or gastrointestinal tract, promoting systemic inflammation and contributing to conditions such as cardiovascular disease, liver cirrhosis, pancreatic cancer, and neurodegenerative disorders [6, 7]. The growing understanding of the human microbiome as a “superorganism” reinforces the idea that microbial communities are integral to human physiology. The Human Microbiome Project has emphasized the significance of these communities in regulating host metabolism, immunity, and disease susceptibility. In this context, the oral microbiome has emerged as a focal point for both disease diagnosis and personalized therapeutic strategies. Its accessibility and responsiveness to external stimuli make it a practical target for non-invasive diagnostics and interventions [3, 7, 8].

Recent interest has turned toward natural products as promising modulators of the oral microbiome. These substances, derived from plants and microorganisms, exhibit antimicrobial, anti-inflammatory, and immunoregulatory properties. Compounds such as polyphenols, flavonoids, and essential oils have shown potential to selectively inhibit pathogenic bacteria while supporting commensal populations [2, 9, 10]. For example, tea extracts have been shown to suppress the growth of harmful genera like *Escherichia*/*Shigella* while promoting beneficial butyrate-producing bacteria such as *Faecalibacterium* and *Coprococcus*. These functional molecules not only modulate microbial composition but also influence host signaling pathways that govern inflammation and tissue homeostasis [10]. Natural products are also being explored for their capacity to prevent or mitigate oral diseases by restoring microbial equilibrium. Probiotic and prebiotic interventions aim to shift the oral microbiome toward a more health-associated state, thus preventing the initiation and progression of periodontitis and other inflammatory conditions. Such interventions hold the potential to indirectly reduce systemic disease risk by maintaining oral health and reducing microbial translocation and systemic inflammation [2, 5]. Because the oral microbiota plays such an important role in overall health and its disbalance between good and bad microorganism cause several illnesses and also natural products show real potential for guiding microbial balance, there is a clear need to bring current evidence together and map out what comes next. This review explores how natural products help shape the oral microbiome, support oral health, and prevent disease. By drawing on recent findings we also discuss the mechanisms through which these compounds interact with and influence oral microbial communities. Ultimately, the aim is to provide insight that can support the development of effective, evidence-based strategies that use natural products to improve both oral and systemic health. The literature for this review was collected from PubMed, Scopus, ScienceDirect, and Google Scholar using keywords such as natural compounds, oral microbiome, health effects, probiotics, safety, oral dysbiosis, and disease. Studies published between 2015 and 2025 were primarily considered, and only a few older references were included when essential for background understanding or specific content needs. All relevant and high-quality sources were selected to support the preparation of this manuscript.

## Structure, composition, and overview of oral microbiota

**Fig. 1:**
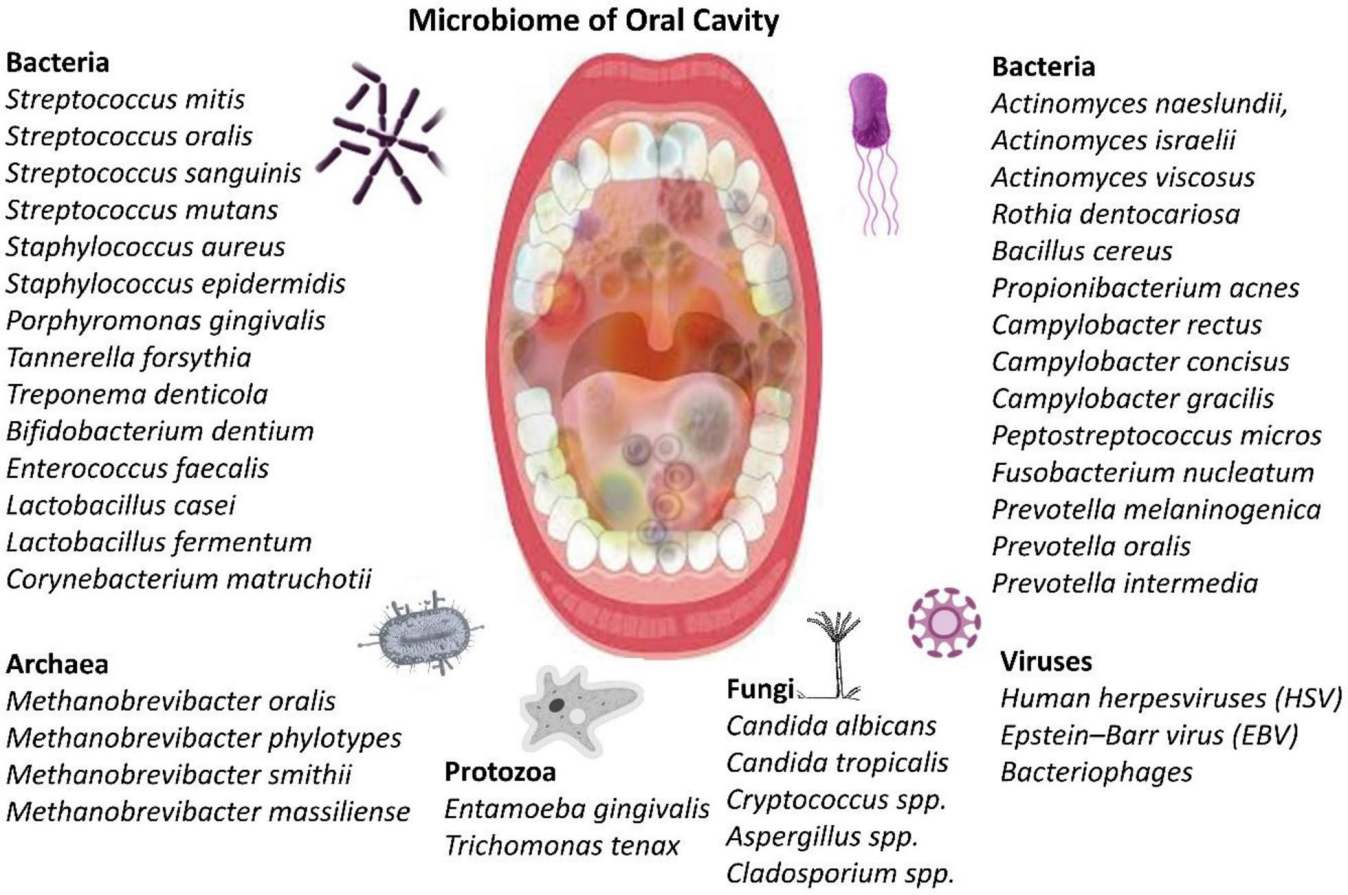
The diagram shows the oral cavity microbiome, comprising bacteria, archaea, fungi, viruses, and protozoa

The oral cavity of hosts supports over 700 microbial species that help in selective adhesion, interbacterial co-aggregation, salivary flow, nutrient availability, and host immune responses [11, 12]. For example, the tongue dorsum supports anaerobic species due to its papillary surface, while the buccal mucosa harbors aerobes owing to higher oxygen exposure. Saliva continuously links these microenvironments, yet the microbial communities maintain distinct structures due to local adhesion forces and micron-scale environmental gradients [12]. A brief overview of microbiome of oral cavity is given in Fig. 1.

Oral microbial communities are established shortly after birth through exposure to environmental and maternal microbes. Infants acquire bacteria through direct contact with caregivers and the environment, and their oral microbiome composition continues to evolve with tooth eruption and dietary changes. *Streptococcus* species, especially *viridans streptococci*, act as early colonizers. As the microbial network matures, more complex biofilms emerge, involving species such as *Fusobacterium nucleatum* that function as bridge organisms, linking early and late colonizers [13]. Advanced sequencing technologies such as 16S rRNA gene sequencing and whole-metagenome shotgun sequencing have transformed the understanding of oral microbiome complexity. These technologies have revealed new phylotypes and enabled functional analyses of microbial communities, even those unculturable by traditional methods [14, 15]. The oral microbiome is now known to be remarkably stable over time in individuals, yet it remains highly personalized due to individual-specific factors such as genetics, age, lifestyle, and health status. Genetic influences on the oral microbiota have been established through studies involving monozygotic and dizygotic twins. Research from the Colorado Twin Registry demonstrated that monozygotic twins have significantly more similar oral microbial profiles than dizygotic twins or unrelated individuals. This similarity persists even after changes in cohabitation, suggesting that certain microbial traits are heritable. Genome-wide association studies (GWAS) have identified genetic loci, including regions near the IMMPL2 gene on chromosome 7 and the INHBA-AS1 gene on chromosome 12, that are significantly associated with the composition of the oral microbiome [16].

Environmental and behavioral factors also significantly shape the oral microbiota. Diet, oral hygiene, antibiotic exposure, and substance use contribute to microbial selection and succession in the mouth. For instance, regular dental care is associated with reduced prevalence of pathogenic species such as *Treponema*, while obesity in youth correlates with higher abundance of certain pathogens like *Treponema* and *Leptotrichia* [17]. Moreover, aging contributes to physiological changes in the oral environment, although studies show that while overall microbiome composition remains relatively stable, certain bacterial genera such as *Fusobacterium*, *Porphyromonas*, and *Parvimonas* increase with age and may serve as biomarkers for health status in elderly populations [18]. The oral microbiota does not act in isolation but interacts continuously with the host immune system. This dynamic equilibrium maintains oral health by preventing colonization by pathogens. However, disruption of this balance, known as dysbiosis, can initiate oral diseases such as dental caries and periodontitis. Caries is associated with acidogenic and aciduric species, while periodontitis involves anaerobic proteolytic bacteria such as *Porphyromonas gingivalis*, *Treponema denticola*, and *Tannerella forsythia*. These pathogens thrive in inflamed, oxygen-poor subgingival environments and manipulate host immune responses, contributing to tissue destruction [19].

The composition of oral microbiota can also reflect and potentially influence systemic health. Table 1 represents key microorganisms in the oral cavity, their types, and health effects. Several studies have identified associations between oral dysbiosis and chronic conditions, including cardiovascular disease, diabetes, obesity, and hypertension. For instance, elderly individuals with hypertension exhibit significant shifts in oral microbiota, with higher levels of *Treponema* and *Leptothrix* and reduced levels of *Actinomyces* and *Capnocytophaga*. These microbial patterns are accompanied by functional changes such as increased degradation of chlorinated compounds, suggesting possible mechanistic roles in disease pathogenesis [20]. Understanding oral microbiota also offers translational potential for personalized medicine. Since the oral cavity is easily accessible, it is feasible to monitor microbiome changes and apply targeted interventions. Microbiome-based diagnostics can help in the early detection of systemic diseases, while microbial modulation strategies, such as probiotics, prebiotics, or precision antimicrobials, may provide novel therapeutic avenues [14, 15]. The integration of sequencing-based technologies and bioinformatics has expanded knowledge of its structure and function, setting the stage for microbiome-driven diagnostics and therapeutics in the near future.

**Table 1:** Key microorganisms in the oral cavity, their types, and health effects

Microorganism	Type	Gram-positive/gram-negative	Health effects	References
*Streptococcus mitis*	Bacterium	Positive	Keystone pioneer colonizer; supports biofilm assembly; modulates immunity and epithelial responses	[21]
*Streptococcus oralis / sanguinis*	Bacterium	Positive	Dominant commensals produce hydrogen peroxide, inhibit pathogens, and may occasionally cause endocarditis and meningitis	[22, 23]
*Streptococcus mutans*	Bacterium	Positive	Major cariogenic species, acidogenic and aciduric, promote enamel demineralization	[24]
*Staphylococcus aureus, S. epidermidis*	Bacterium	Positive	Occasional oral colonizers; implicated in opportunistic infections (e.g., abscess, endocarditis)	[25, 26]
*Porphyromonas gingivalis*	Bacterium	Negative	Keystone periodontal pathogen (“red complex”); immune subversion; systemic links (CVD, RA, IBD)	[27]
*Tannerella forsythia*	Bacterium	Negative	Part of the red complex; implicated in periodontitis and tissue destruction	[28]
*Treponema denticola*	Bacterium	Negative	Spirochete in red complex; associated with periodontal disease and systemic inflammation	[29]
*Bifidobacterium dentium*	Bacterium	Positive	Aciduric microbiota member; contributes to caries progression	[30]
*Enterococcus faecalis*	Bacterium	Positive	Associated with persistent endodontic infections, biofilm-forming	[31]
*Lactobacillus casei & L. fermentum*	Bacterium	Positive	Aciduric, linked to caries progression; also, probiotic candidates	[32]
*Corynebacterium matruchotii*	Bacterium	Positive	Involved in healthy plaque architecture; abundant in supragingival biofilms	[33]
*Actinomyces naeslundii, A. israelii, A. viscosus*	Bacterium	Positive	Early colonizers, roles in root-surface caries and plaque formation	[34]
*Rothia dentocariosa*	Bacterium	Positive	Associated with health; occasionally opportunistic in endocarditis	[35]
*Bacillus cereus*	Bacterium	Positive	Environmental transient; a rare oral opportunist in immunocompromised hosts	[36]
*Propionibacterium acnes*	Bacterium	Positive	Transiently present; implicated in endodontic infections	[37]
*Campylobacter rectus, C. concisus, C. gracilis*	Bacterium	Negative	Associated with periodontitis, inflammatory and systemic links	[38]
*Peptostreptococcus micros*	Bacterium	Positive	Involved in periodontal/abscess infections; frequent in deep biofilms	[39]
*Fusobacterium nucleatum*	Bacterium	Negative	Oral commensal and a periodontal pathogen; Bridge organism; promotes red-complex growth; systemic cancer link	[40]
*Prevotella melaninogenica, P. oralis, P. intermedia*	Bacterium	Negative	Anaerobic commensals on oral mucosae and in dental plaques from early life onwards; In periodontal disease; P. intermedia associated with pregnancy gingivitis	[41]
*Candida albicans*	Fungus (Yeast)	NA	Opportunistic pathobiont; forms biofilm; synergizes with bacteria in caries and periodontitis; main cause of oral candidiasis	[42, 43]
*Candida tropicalis*	Fungus (Yeast)	NA	Less common; associated with caries and mucosal lesions in immunocompromised hosts; morphogenesis similar to C. albicans	[43, 44]
*Cryptococcus spp.*	Fungus (Yeast)	NA	Present in normal oral mycobiome; potential opportunistic in compromised hosts	[45]
*Aspergillus spp.*	Fungus (Mold)	NA	Present in healthy and diseased mouths; can exacerbate periodontitis via biofilm interactions; opportunistic in immunocompromised patients	[46]
*Cladosporium spp.*	Fungus (Mold)	NA	Frequently detected; environmental; generally non-pathogenic, but may affect mucosal immunity	[47]
*Entamoeba gingivalis*	Amoeboid protozoan	NA	Present in 77–95% of periodontal pockets; induces inflammation via IL-8, TNF-α, IL-1β; feeds on neutrophils and epithelial cells, contributing to periodontal tissue destruction	[48]
*Trichomonas tenax*	Flagellated protozoan	NA	Found in ~ 56–70% of periodontitis cases; secretes proteases and phosphatases that degrade extracellular matrix and bone tissue; exacerbates periodontal inflammation	[48]
*Methanobrevibacter oralis*	Archaea	NA	Methanogen enriched in periodontitis supports anaerobic fermentation and microbial shifts	[49]
*Methanobrevibacter phylotypes*	Archaea	NA	Methanogen enriched in periodontitis supports anaerobic fermentation, methanogenesis, and microbial shifts	[50]
*Methanobrevibacter smithii*	Archaea	NA	Methanogen enriched in periodontitis supports anaerobic fermentation, methanogenesis, and balance microbial community	[51]
*Methanobrevibacter massiliense*	Archaea	NA	Associated with various oral pathologies like periodontitis and endodontic infections, Interacts with other oral bacteria	[51]
*Human herpesviruses (HSV), Epstein–Barr virus (EBV)*	Virus	NA	Causes oral lesions; may exacerbate periodontal disease and local immune changes; carcinoma	[52]
*Bacteriophages*	Virus	NA	Regulate bacterial population; influence biofilm dynamics	[53]

## Oral microbiome dysbiosis drives systemic disease

**Fig. 2:**
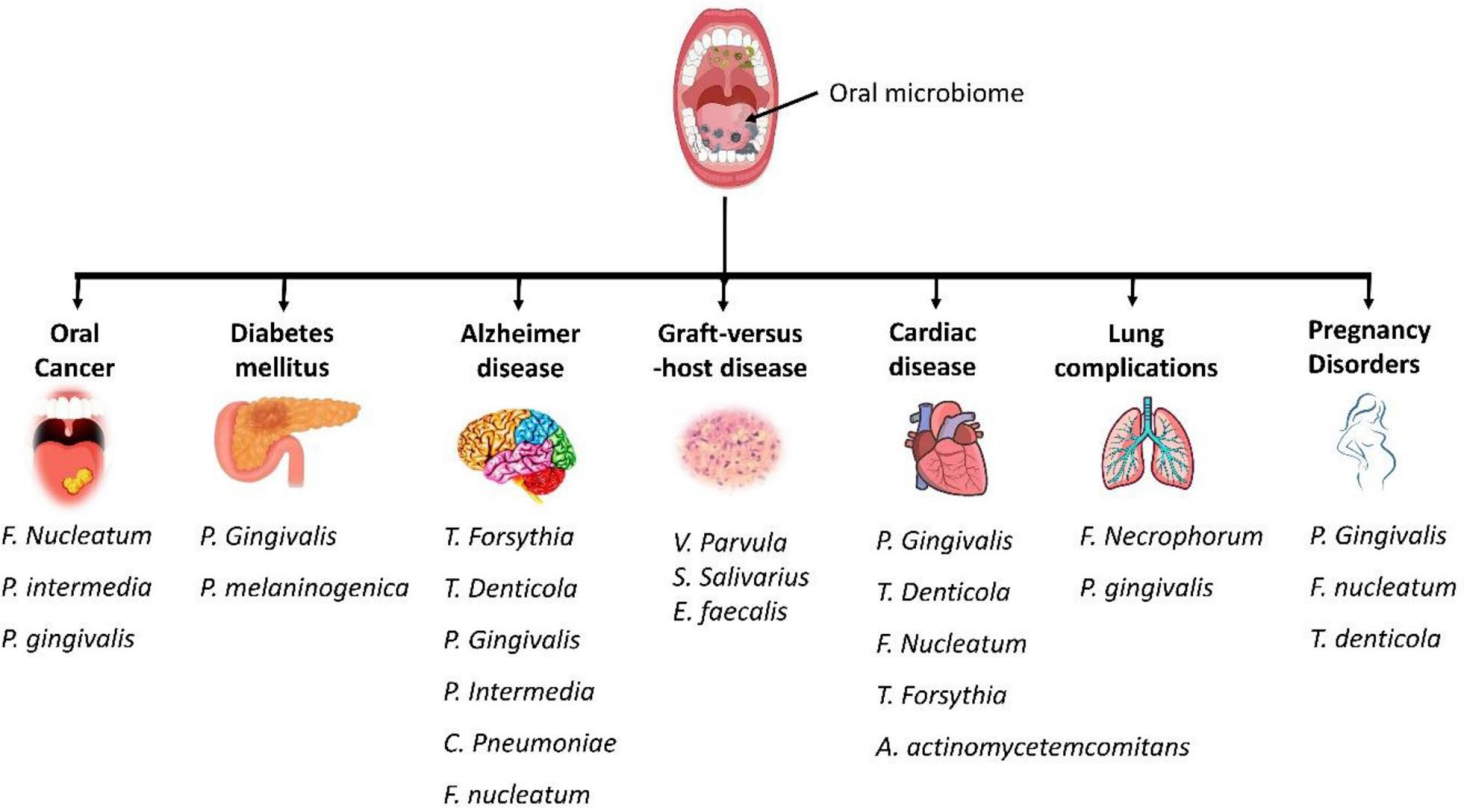
Oral dysbiosis linked disorders and systemic disease. The image shows the microorganism involved in different disorders and diseases

Oral dysbiosis refers to an imbalance in the microbial community that inhabits the mouth. It can result from factors such as poor oral hygiene, dietary habits, medication use, and disease. Dysbiosis manifests as a rise in pathogenic bacterial populations alongside a reduction in beneficial species. There is strong evidence linking oral dysbiosis to the onset and progression of dental caries, periodontitis, and oral cancers [54, 55]. It also shows emerging associations with systemic diseases like cardiovascular disease and diabetes. These observations drive efforts to restore microbial homeostasis using prebiotic, probiotic, and antimicrobial strategies. Dental caries arises when frequent consumption of fermentable sugars fuels acidogenic bacteria such as *Streptococcus mutans*, *Lactobacillus, Actinomyces*, and *Bifidobacterium*. Their acid production lowers plaque pH, triggering enamel demineralization. Periodontitis reflects a dysbiotic shift toward anaerobic proteolytic bacteria, including *Porphyromonas gingivalis*, *Tannerella forsythia*, and *Treponema denticola*. These pathogens secrete virulence factors that disrupt host immune responses, destroy periodontal tissues, and alter host metabolism [54, 56]. A detailed systemic disease or complications derived from oral dysbiosis are given in Fig. 2 and detailed below:

### Lung complications

It is increasingly evident that oral bacteria contribute to lung-related diseases through inflammatory and immune-modulating mechanisms. *Porphyromonas gingivalis*, a key periodontal pathogen, has been implicated in aspiration pneumonia, where it activates innate immune responses via TLR2. In a mouse model, deficiency of TLR2 led to reduced production of inflammatory mediators such as TNF-α, IL-6, IL-12p70, NO, and impaired bacterial clearance in the lungs. It was shown that alveolar macrophages, particularly reliant on NO production, were essential in bacterial killing, highlighting the importance of local immune function [57]. *Fusobacterium necrophorum*, another oral bacterium, was identified as the sole cause of lung nodules in a healthy individual, indicating its potential to cause parenchymal lung disease independent of prior illness [58]. Additionally, clinical data show that individuals with periodontitis are nearly three times more likely to develop nosocomial pneumonia compared to those with healthy gums, even after adjusting for confounding factors. These findings underscore a strong link between oral infections and lung complications, particularly in vulnerable populations [59].

### Cardiovascular disease

There are documented links between periodontal dysbiosis and stroke. Periodontal pathogens contribute to atherosclerosis, hypertension, and endothelial dysfunction, increasing stroke risk [60]. There is strong scientific evidence linking oral bacteria to cardiovascular disease through mechanisms involving inflammation, immune activation, and direct vascular colonization. It has been shown that *Porphyromonas gingivalis*, a key periodontal pathogen, can enter the bloodstream during chronic periodontitis and induce platelet aggregation through its fimbriae and outer membrane vesicles, potentially contributing to thromboembolic events and atherosclerosis. It was also observed that only *P. gingivalis*, among several tested oral pathogens, had this potent platelet-aggregating activity [61].

It is known that individuals with elevated serum antibodies to *P. gingivalis* and *Aggregatibacter actinomycetemcomitans* show increased prevalence of coronary heart disease (CHD), with combined antibody responses being significantly associated with CHD and lower HDL cholesterol levels [62]. Experimental studies further confirmed that chronic oral infection with *P. gingivalis* in hyperlipidemic mice led to aortic plaque formation, systemic inflammation, and detection of the bacteria in both oral tissues and aorta, indicating bacterial dissemination and immune response [63]. *Porphyromonas gingivalis*, an oral pathogen linked to gum disease, can trigger systemic inflammation and contribute to heart disease, particularly atherosclerosis. It produces atypical lipid A structures in its lipopolysaccharide (LPS) that manipulate the host’s immune response by evading Toll-like receptor 4 (TLR4) detection. Antagonistic lipid A enables bacterial survival in macrophages, leading to chronic low-grade inflammation and vascular damage. In mouse models, this immune evasion accelerates atherosclerosis. Conversely, lipid A variants that activate TLR4 lead to stronger immune responses but reduced bacterial survival. These findings highlight how *P. gingivalis* drives chronic inflammation and heart disease through immune evasion strategies [64]. It was found that *Treponema denticola* infection also causes loss of alveolar bone and significantly enhances aortic plaque area, alongside increased oxidized LDL and reduced nitric oxide levels [65]. There is further support from endarterectomy studies, where bacterial DNA from *P. gingivalis*, *A. actinomycetemcomitans*, *Tannerella forsythia*, and *Fusobacterium nucleatum* was detected in carotid atheromatous plaques [66]. These findings collectively suggest a causal role of oral bacteria in cardiovascular pathology.

### Diabetes mellitus

Periodontitis and diabetes mellitus share a bidirectional relationship that is driven by inflammation, immune dysregulation, and metabolic disturbances. It is well established that diabetes, particularly when poorly controlled, significantly increases the risk and severity of periodontitis by approximately threefold [67]. Hyperglycemia impairs neutrophil function and promotes the overproduction of proinflammatory cytokines such as tumor necrosis factor-α (TNF-α), interleukin-1, and fibrinogen, all of which contribute to periodontal tissue breakdown [68]. In turn, chronic periodontal inflammation leads to elevated systemic inflammatory mediators that can worsen insulin resistance and disrupt glycemic control in diabetic individuals [69]. Evidence shows that individuals with poor metabolic control exhibit higher prevalence and severity of periodontitis compared to those with good control or healthy individuals, along with elevated levels of TNF-α and reduced tooth count [68]. Obesity compounds this interaction by increasing adipocyte-derived TNF-α, which further interferes with insulin signaling and promotes periodontal inflammation [70]. The presence of severe periodontitis in diabetics is also associated with increased risks of macroalbuminuria, end-stage renal disease, and cardiorenal mortality. Treatment of periodontal disease has been shown to improve glycemic control, reflected by reductions in HbA1c levels by about 0.4%, supporting the clinical relevance of oral care in diabetes management [67]. It is clear that inflammation serves as the central mechanistic link between these two diseases, and integrating periodontal therapy into diabetes care strategies may provide systemic health benefits [69]. There is growing evidence that oral microbial changes are linked to type 2 diabetes mellitus (T2DM), even without oral disease. It was found that *Porphyromonas gingivalis* and *Prevotella melaninogenica* were significantly enriched in T2DM patients, while *Streptococcus mutans* and *Streptococcus sobrinus* levels remained unchanged. It was also observed that salivary levels of cadaverine and *L*-( +)-leucine, as well as plaque levels of N-acetyldopamine and 3,4-dimethylbenzoic acid, were significantly higher in T2DM patients. There appears to be a relationship between oral microbes and harmful metabolite dysregulation, suggesting a role for oral microbiota in T2DM and potential biomarkers for early disease detection [71].

### Neurodegenerative disease

Emerging evidence also supports the idea of an oral–brain axis in neuropsychiatric disorders. Translocated oral bacteria may reach the brain via the bloodstream or neural pathways, triggering inflammation and contributing to conditions like depression, anxiety, Parkinson’s disease, and dementia. Although underlying mechanisms remain under investigation, preliminary findings have sparked interest in how oral dysbiosis may impact brain health [72]. Periodontitis is a chronic inflammatory disease driven by specific oral pathogens, and its systemic impact has been increasingly linked to Alzheimer’s disease (AD), a neurodegenerative disorder marked by cognitive decline and neuroinflammation. There is growing evidence suggesting that systemic inflammation originating from periodontal infection may contribute to the pathogenesis or progression of AD [73–75]. Studies have shown that serum immunoglobulin G antibodies against periodontal bacteria, such as *Fusobacterium nucleatum* and *Prevotella intermedia*, were significantly elevated in individuals who later developed AD, even when they were cognitively normal at baseline [73]. These elevated antibody levels imply a chronic immune response to oral pathogens, which may precede cognitive decline.

It has also been demonstrated that plasma levels of tumor necrosis factor-alpha (TNF-α), a proinflammatory cytokine, are elevated in AD patients and correlate with the presence of antibodies to periodontal bacteria, reinforcing the role of systemic inflammation in AD diagnosis [76, 77]. Notably, animal studies provide mechanistic insight into this relationship. In a mouse model deficient in apolipoprotein E, oral infection with pathogens such as *Porphyromonas gingivalis*, *Treponema denticola*, *Tannerella forsythia*, and *F. nucleatum* led to bacterial DNA being detected in brain tissues and activation of the complement system, causing neuronal injury [78]. Furthermore, the detection of lipopolysaccharide (LPS), a virulence factor from *P. gingivalis*, in human AD brain tissues suggests that components of oral bacteria can translocate and persist in the brain, promoting local inflammation [79]. Histological analyses have shown co-localization of *Chlamydia pneumoniae* with amyloid plaques in AD brain regions, indicating that infection-related inflammation may intensify amyloid deposition [80]. Complementary findings have confirmed that the same inflammatory mediators elevated in periodontitis such as interleukin-6 and TNF-α are significantly present in AD patients with coexisting periodontitis, further supporting the notion of a shared inflammatory axis [77, 81]. Neuroinflammation is a defining feature of AD, characterized by microglial activation and sustained expression of proinflammatory cytokines and complement proteins, which contribute to a cycle of neuronal damage [82, 83]. Thus, the chronic inflammatory burden imposed by periodontitis appears to act as a peripheral trigger that may amplify neuroinflammatory pathways in the brain, establishing a plausible biological link between these two prevalent diseases [74, 75].

### Pregnancy disorders

Periodontal disease, a chronic inflammatory condition affecting the supporting structures of teeth, is increasingly recognized as a contributing factor to adverse pregnancy outcomes, including preterm birth, low birth weight, pre-eclampsia, and fetal loss [84]. Evidence supports that oral pathogens and their products can disseminate systemically and translocate to the maternal–fetal interface, where they may provoke local inflammation and immune responses [85]. The migration of *Fusobacterium nucleatum*, a key periodontal pathogen, from the maternal oral cavity to the placenta has been documented in both clinical and case-based studies. In one case, this organism was directly isolated from the placenta and fetal tissues in a stillbirth incident, with genetic analysis confirming its origin in the mother’s subgingival plaque [86]. Another study demonstrated a 100% sequence identity between *F. nucleatum* in the amniotic fluid and strains from the maternal or paternal oral microbiota, suggesting hematogenous transmission from oral reservoirs [87].

Moreover, *Porphyromonas gingivalis*, another major periodontal pathogen, has been identified in the placental tissues of women with chorioamnionitis, with its antigens found in trophoblastic and vascular cells. The presence of *P. gingivalis* was significantly higher in these women compared to those with normal-term pregnancies, indicating a strong association with infection-driven preterm delivery [88]. Periodontal infection may also contribute to fetal resorption through molecular mimicry mechanisms. Antibodies induced by *P. gingivalis* infection have been shown to cross-react with β2-glycoprotein I, a molecule implicated in fetal loss, enhancing the risk of pregnancy complications [89].

Toll-like receptors (TLRs), particularly TLR-2 and TLR-4, mediate innate immune responses at the maternal–fetal interface. Their increased expression in placental tissues of women with hypertensive pregnancy disorders coincides with the detection of periodontal pathogens such as *T. denticola* and *P. gingivalis*, linking periodontal inflammation with placental immune activation and adverse outcomes [90, 91]. Preventive and therapeutic interventions during pregnancy have shown potential to mitigate these risks. Non-surgical periodontal therapy, combined with intensive oral hygiene practices, significantly reduced inflammatory markers like IL-1β and TNF-α in gingival crevicular fluid and improved periodontal health, although its direct effect on birth outcomes requires further investigation [92]. Still, the limited availability of prenatal oral health interventions focused on maternal oral status underscores the need for integrated, evidence-based strategies to protect maternal and fetal health [93].

### Graft-versus-host disease (GVHD)

The oral cavity plays a critical role in the pathogenesis and clinical presentation of chronic graft-versus-host disease (cGVHD), a major complication of allogeneic hematopoietic cell transplantation (alloHCT). It is affected in up to 83% of patients who develop cGVHD and contributes significantly to post-transplant morbidity and decreased quality of life [94]. The oral manifestations of cGVHD include mucosal lesions resembling lichen planus, erythema, ulcerations, superficial mucoceles, salivary gland dysfunction causing xerostomia, and perioral fibrosis leading to trismus [95, 96]. These symptoms often result in pain, poor oral intake, malnutrition, and risk of secondary infections such as candidiasis or even oral squamous cell carcinoma [97, 98]. Diagnosis relies on clinical features supported by histopathological examination of oral mucosa and minor salivary glands [97]. Immunologically, the condition is driven by donor-derived inflammatory T cells and lymphocyte-mediated damage to oral tissues, with some cases requiring long-term immunosuppression [96]. In a study, there was expansion of *Streptococcus salivarius* and *Veillonella parvula* in oral cGVHD, with oral/gut microbiota overlap suggesting ectopic colonization and a potential role in disease pathogenesis [99]. It has also been observed that oral dysbiosis, triggered by mucositis or oral complications post-HCT, exacerbates cGVHD. Increased colonization by *Enterococcaceae* (*E. faecalis*) and translocation to lymphatic and gastrointestinal tissues stimulates systemic inflammation through activation of antigen-presenting cells and expansion of inflammatory T cells. Importantly, modulation of the oral microbiota during transplantation has shown potential in mitigating disease severity. Current treatment focuses on symptom management using topical corticosteroids or calcineurin inhibitors, but challenges remain in preventing long-term tissue damage and secondary malignancies. Multidisciplinary care involving dental professionals is essential to monitor and manage the complex oral health requirements of alloHCT survivors [96, 100].

### Opportunistic infections

It is well recognized that immunosuppression significantly disrupts the oral microbiome, leading to dysbiosis and increasing susceptibility to opportunistic infections. It alters the immune surveillance in the oral cavity, particularly by depleting CD4 + T lymphocytes, which are critical for microbial control and mucosal immunity [101]. It leads to an imbalance between commensal and pathogenic microbes, where beneficial genera like *Streptococcus* and *Veillonella* are reduced, while opportunistic pathogens such as *Prevotella*, *Campylobacter*, *Megasphaera*, and *Veillonella* species increase, particularly in untreated HIV patients. There is also reduced colonization of *Neisseria flavescens* in patients undergoing antiretroviral therapy, suggesting that therapy itself reshapes the microbial composition [102]. It has been observed that immunocompromised children, both HIV-infected and HIV-exposed but uninfected, display distinct oral microbial communities compared to healthy children, with alterations more pronounced in individuals with low CD4 counts [103]. It indicates that immune suppression, rather than HIV status alone, plays a primary role in microbial imbalance. There is persistent dysbiosis in HIV-infected individuals despite antiretroviral therapy, implicating immune cell dysfunction and altered salivary composition as key contributors [101].

There are experimental models where corticosteroid-induced immunosuppression facilitates biofilm formation of *Candida albicans*, resulting in tissue invasion and dissemination into systemic organs. It promotes co-colonization by *Enterococcus faecalis*, a common gut-derived opportunist, exacerbating systemic dysbiosis [104]. It further supports that oral immunosuppression can drive microbial translocation beyond the oral cavity, worsening systemic health. There is growing interest in exploring probiotic interventions to rebalance the oral microbiome under immunosuppressive conditions, although clinical evidence remains limited [101]. It becomes essential to understand and manage oral dysbiosis in immunosuppressed individuals to prevent oral and systemic complications, highlighting the need for integrated therapeutic strategies.

### Carcinogenesis

Oral dysbiosis also contributes to carcinogenesis through multiple molecular mechanisms. Bacterial overgrowth can lead to chronic inflammation and DNA damage. Microbial metabolites, such as reactive oxygen and nitrogen species, serve as pro-carcinogenic agents. Some bacteria inhibit local immunity and shape a tumor-favorable microenvironment. Associations have been observed between oral dysbiotic profiles and oral squamous cell carcinoma, as well as colorectal cancer, where oral microbiome-based biomarkers display diagnostic potential [55, 105–107]. Systemic impacts of oral dysbiosis occur through microbial translocation and immune-mediated pathways. Oral bacteria can enter systemic circulation or be swallowed, reaching the gastrointestinal tract. This translocation has been documented in multiple disorders, including inflammatory bowel disease, celiac disease, and colorectal cancer. Clinical studies have shown that colonization of the oral microbiome in the gut is linked with systemic diseases [108].

There is growing scientific evidence supporting a strong association between periodontal disease and the development of oral squamous cell carcinoma (OSCC). Periodontitis, a chronic inflammatory condition driven by pathogenic bacteria such as *Porphyromonas gingivalis* and *Fusobacterium nucleatum*, contributes to tumorigenesis through multiple molecular pathways. It promotes OSCC by activating the IL-6-STAT3 signaling cascade and interacting directly with oral epithelial cells via Toll-like receptors [109]. *P. gingivalis* enhances OSCC invasiveness by increasing proMMP9 expression and activating it via gingipain proteases. This mechanism is mediated by ERK1/2-Ets1, p38/HSP27, and PAR2/NF-κB signaling pathways. It also selectively affects highly invasive cancer cells, suggesting its role in aggressive tumor progression [110]. The relevance of this connection is further reinforced by population-based studies. Data from Taiwan revealed that patients with periodontitis had a 1.79-fold higher risk of developing oral cancer compared to those with gingivitis [111]. Additionally, large-scale epidemiological reviews and meta-analyses consistently report elevated oral cancer risk in individuals with periodontal disease, with odds ratios reaching up to 3.53 [112]. In another meta-analysis, *Porphyromonas gingivalis* and *Prevotella intermedia* have shown an increased incidence of cancer risk. These findings highlight the systemic impact of periodontal pathogens and support the need for integrated oral health strategies in cancer prevention efforts [113, 114].

## The natural product, types, and their effects on oral microbiota

**Fig. 3:**
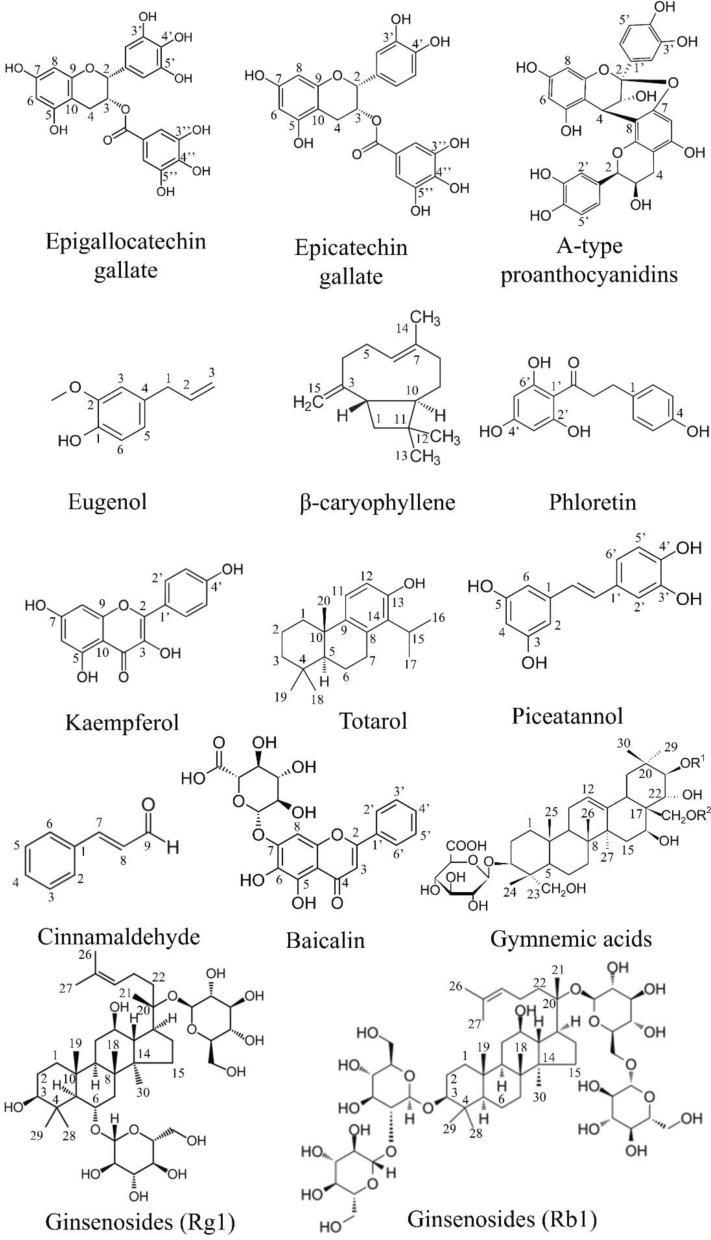
Natural compound against oral microbiome

**Fig. 4:**
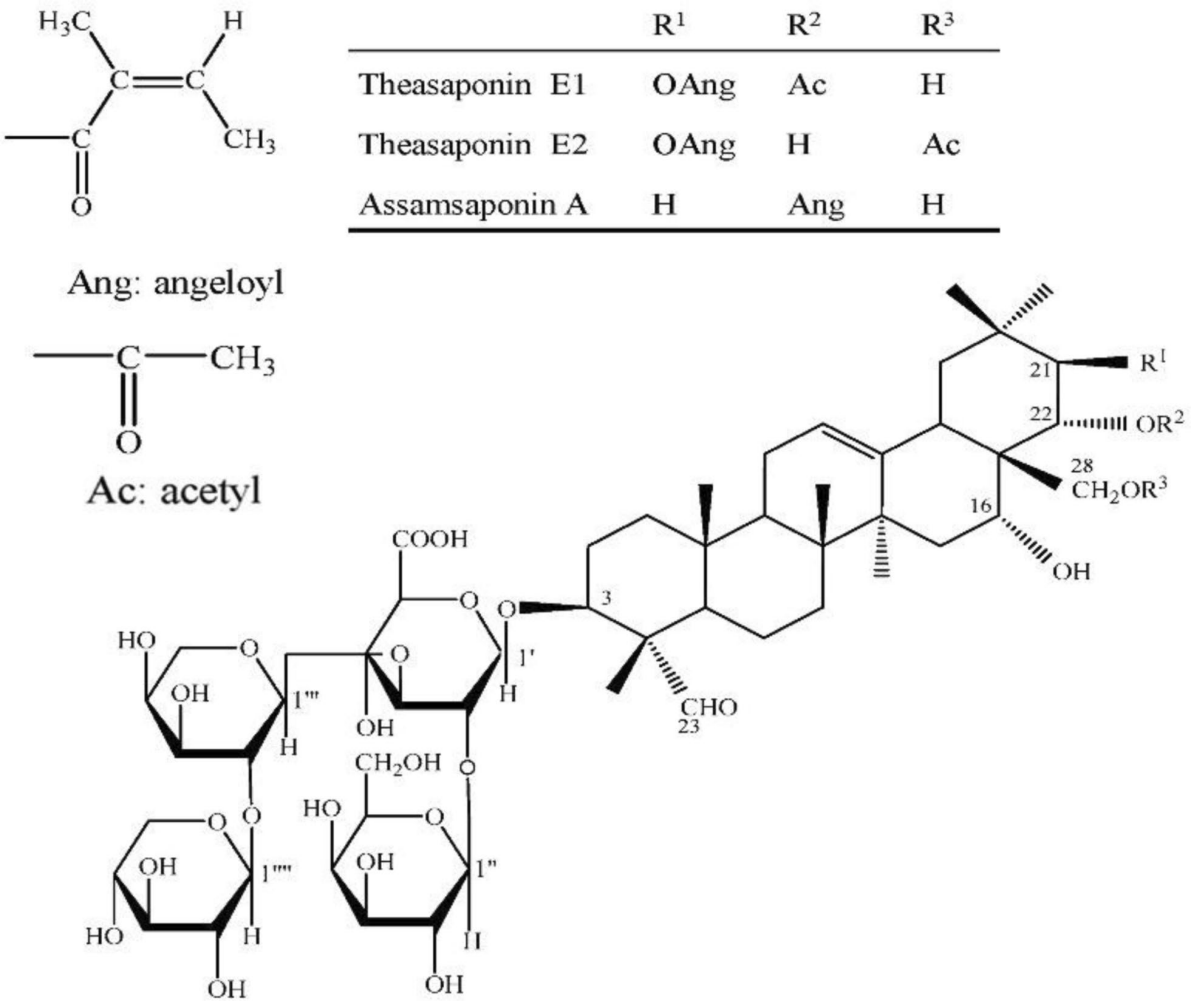
Tea saponins assamsaponin A (ASA), theasaponin E1 (TE1), and theasaponin E2 (TE2)

Natural products are biologically active compounds derived primarily from plants, microbes, and other natural sources. These compounds have been employed for centuries in traditional medicine and are now recognized for their immense potential in modern therapeutic applications, including oral healthcare. Among their key roles is the ability to interact with the oral microbiota, the complex microbial community inhabiting the human oral cavity. These natural products possess properties such as antimicrobial, antioxidant, and anti-inflammatory activities, which allow them to modulate microbial composition and maintain oral health [16, 115]. Plant-derived natural products are primarily secondary metabolites, which are not essential for the basic survival of the plant but serve critical ecological functions. These include alkaloids, flavonoids, polyphenols, terpenes, saponins, quinones, coumarins, glycosides, carotenoids, and tannins [115, 116]. Each of these compounds exhibits unique biological activities that influence microbial growth and survival (Refer to Fig. 3 and Fig. 4). For example, polyphenols and alkaloids have demonstrated significant antimicrobial effects against oral pathogens such as *Streptococcus mutans* and *Lactobacillus species*, which are commonly associated with dental caries [117, 118]. Acidogenic and acid-tolerant bacteria metabolize dietary carbohydrates into acids, leading to enamel demineralization and cavity formation. The main culprits include *Streptococcus mutans*, *Lactobacillus*, *Actinomyces*, *Bifidobacterium*, and *Streptococcus* species [117].

Polyphenols are a broad group of plant-based compounds that are commonly found in dietary sources such as green tea, grapes, and berries. These compounds exhibit potent antioxidant properties and contribute to oral health by neutralizing reactive oxygen species, reducing inflammation, and inhibiting bacterial adherence and biofilm formation. In the oral cavity, polyphenols can modulate bacterial gene expression, suppress acid production, and prevent the progression of dental caries [119, 120]. The chemical structure of polyphenols allows them to interact with bacterial cell membranes and interfere with quorum sensing, which is essential for biofilm maturation [118]. Epigallocatechin gallate (EGCG), a polyphenolic catechin from green tea (*Camellia sinensis*), suppresses the growth, adhesion, and biofilm formation of *Porphyromonas gingivalis* and *Streptococcus mutans* by disrupting bacterial membranes, inhibiting key virulence enzymes such as gingipains and glycosyltransferases, neutralizing oxidative stress, and reducing inflammation through inhibition of MMP-9 [121, 122]. Piceatannol, a stilbene found in *Rhus species* and grapes, inhibits *Streptococcus mutans* glucosyltransferases, resulting in reduced extracellular polysaccharide production and lower biofilm density [123]. Other polyphenols epicatechin gallate (ECG) found in green tea interferes with the production of bacterial virulence enzymes, enhances the activity of conventional antibiotics through synergistic effects, and helps reverse methicillin-resistant *Staphylococcus aureus* (MRSA) resistance by inhibiting bacterial efflux pump mechanisms [124]. Similarly, A-type proanthocyanidins (PACs), a polyphenolic found in cranberry extracts, inhibit *Streptococcus mutans* glucosyltransferase activity, thereby reducing acid production and limiting biofilm formation in the oral cavity [125]. Phloretin a polyphenolic dihydrochalcone abundant in apples, disrupts *Staphylococcus aureus* and *Escherichia coli* biofilms by suppressing toxin and adhesin expression, while modulating efflux pumps to limit early bacterial colonization [126, 127].

Terpenoids, another significant class of natural products, are present in essential oils derived from plants like thyme, clove, and eucalyptus. These compounds exhibit a range of pharmacological activities, including antimicrobial, antioxidant, and anti-inflammatory properties [128, 129]. Terpenoids, such as monoterpenoids and sesquiterpenoids, disrupt bacterial membrane integrity, leading to cell lysis and death. Their use in oral hygiene products has gained popularity due to their effectiveness against a wide spectrum of oral pathogens and their relatively low toxicity [128].

Eugenol, a phenylpropanoid terpene from clove oil (*Syzygium aromaticum*), disrupts bacterial and fungal membranes, inhibits ATPase and proteases, and induces oxidative stress [130]. Also, other compound *β*-caryophyllene a known sesquiterpene present in clove and other essential oils, enhances microbial membrane permeabilization and exhibits strong synergistic antibacterial activity when combined with conventional antibiotics [131]. Totarol, a phenolic diterpene derived from gymnosperm wood such as *Podocarpus* species, disrupts Gram-positive bacterial membranes and is effective against *S. mutans, E. faecalis*, and *P. acnes*, with notable antibiotic synergy [132]. Saponins are naturally foaming agents when mixed with water. It occurs naturally and test bitter. Ginsenosides (Rg1, Rb1, and related compounds) are triterpenoid saponins from roots of *Panax ginseng* reduce cell surface hydrophobicity and extracellular polysaccharide production in *P. gingivalis* and *F. nucleatum*, thereby weakening bacterial adhesion and biofilm formation [133]. Tea saponins (ASA, TE1, and TE2) isolated from seeds of *Camellia sinensis* inhibit *Candida albicans* adhesion, biofilm development, and the yeast-to-hyphae transition, which is critical for fungal virulence [134]. Gymnemic acids which is a triterpenoid saponins derived from leaves of *Gymnema sylvestre* suppress *C. albicans* hyphal growth and disrupt mixed biofilms formed with *Streptococcus gordonii*, reducing fungal–bacterial cooperation in oral biofilms [135].

Alkaloids are nitrogen-containing compounds that serve as the basis for many pharmacologically active drugs. These molecules exhibit antimicrobial effects by intercalating into DNA and interfering with microbial protein synthesis. In the context of oral health, alkaloids have shown efficacy against cariogenic and periodontal pathogens. Their use in oral care formulations is of particular interest in regions with limited access to conventional dental treatments [116, 118]. Berberine, a plant alkaloid obtained from *Coptidis rhizoma*, has shown strong inhibitory effects on key periodontal bacteria such as *Porphyromonas gingivalis* and *Actinobacillus actinomycetemcomitans*. Beyond suppressing bacterial growth, berberine can reduce the activity of collagen-degrading enzymes (collagenases) produced by these pathogens, thereby limiting tissue destruction. It also functions as an efflux pump inhibitor, which may enhance its antimicrobial effectiveness and help overcome bacterial resistance mechanisms [136]. Another well-studied alkaloid source is *Sanguinaria canadensis* L., which contains a mixture of benzophenanthridine alkaloids collectively known as sanguinaria extract. Among these, sanguinarine is widely incorporated into commercial toothpastes and mouthwashes. Sanguinarine exhibits broad-spectrum antimicrobial and antibiofilm activity against plaque-forming bacteria. In vitro evidence suggests that its antiplaque effect is largely attributed to interference with bacterial adhesion to the newly formed salivary pellicle, thereby preventing early stages of dental biofilm development [137].

Gurmarin, a cyclic peptide isolated from *Gymnema sylvestre*, exhibits a unique antibiofilm action by inhibiting *Staphylococcus aureus* biofilm formation without directly killing the bacterial cells, highlighting its potential as an anti-virulence agent rather than a conventional antimicrobial [138]. Cinnamaldehyde, a phenylpropanoid compound derived from cinnamon bark, effectively disrupts biofilm development by interfering with quorum sensing pathways. In addition, it impairs bacterial motility and suppresses the expression of key virulence factors, thereby reducing pathogenicity without exerting strong selective pressure for resistance [139]. Kaempferol and baicalin are flavonoids derived from *Scutellaria baicalensis* that exhibit strong anti-virulence activity against oral pathogens. These compounds inhibit key adhesion- and colonization-related proteins such as sortase A, SpaP, and glucan-binding proteins in *Streptococcus mutans*, *Enterococcus faecalis*, and *Staphylococcus aureus*, thereby reducing bacterial attachment, biofilm formation, and overall pathogenicity [140]. Cyanidin is flavonoid present in red berries, grapes, plums, and vegetables like purple corn and red cabbage. Cyanidin suppresses *Streptococcus mutans* biofilm development at higher concentrations without killing the bacteria. It reduces surface-attached biomass and exopolysaccharide production by limiting water-insoluble glucan synthesis through inhibition of glucosyltransferases GtfB and GtfC [141].

**Fig. 5:**
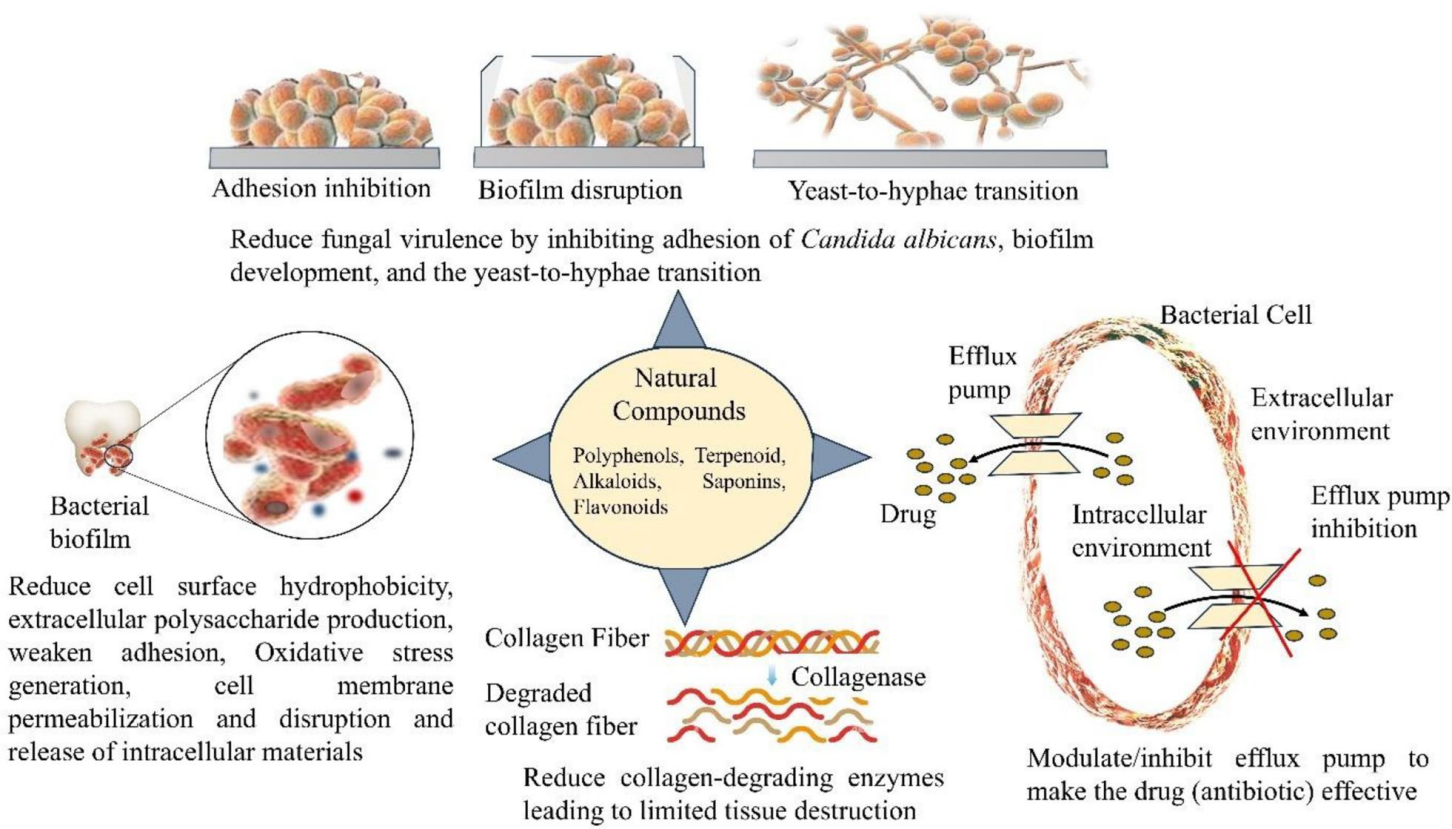
Mechanism of action of different natural compounds against microorganisms. Natural compounds can target microorganisms in several ways. They may inhibit or suppress microbial growth, disrupt cell membranes, or interfere with quorum sensin. Some compounds also prevent biofilm formation, show anti-cariogenic (anti-tooth-decay) activity, reduce acid production, and help protect against enamel demineralization

Figure 5 represents the mechanism of action of different natural compound. Natural products influence the oral microbiota by acting as selective substrates or growth enhancers for beneficial bacteria. For example, certain polyphenols and glycosides serve as fermentation substrates for commensal oral microbes, promoting the growth of species that contribute to a healthy microbial balance. By supporting a diverse and symbiotic microbial community, natural compounds help prevent the overgrowth of pathogenic species and reduce the risk of disease progression [115]. Several traditional and widely used medicinal plants provide bioactive compounds that are being explored for their oral health benefits. Notable examples include *Camellia sinensis* (green tea), *Punica granatum* (pomegranate), *Matricaria recutita* (chamomile), and *Salvadora persica* (miswak). These plants contain a range of phytochemicals with proven antimicrobial and anti-inflammatory activities. Green tea polyphenols, for instance, inhibit bacterial glucosyltransferases, reducing plaque formation and acid production [120, 142]. Essential oils from medicinal plants, which are rich in terpenoids and other volatile compounds, are also valuable in maintaining oral hygiene. Their incorporation into mouthwashes and toothpastes has demonstrated efficacy in reducing plaque, gingival inflammation, and halitosis. The widespread interest in essential oils is driven not only by their therapeutic properties but also by their natural origin and consumer preference for chemical-free alternatives [128, 142]. Commercially available oral care products (toothpaste and powder, mouthwash) of different brands increasingly incorporate natural products and plant extracts to meet consumer demand for safer and more holistic approaches to oral health. There is growing use of plant-derived ingredients such as green tea polyphenols, neem (*Azadirachta indica*), clove oil, miswak (*Salvadora persica*), chamomile, and aloe vera in toothpastes, mouthwashes, and gels due to their antimicrobial and anti-inflammatory properties. The inclusion of essential oils rich in terpenoids, such as thymol, eucalyptol, and menthol, helps reduce dental plaque, control halitosis, and improve gingival health. It is well recognized that polyphenols and flavonoids inhibit biofilm formation and suppress virulence factors of cariogenic and periodontal pathogens. There is also increasing interest in herbal formulations containing probiotics, xylitol, and plant-based antioxidants to support a balanced oral microbiome. The commercial success of these products reflects both traditional knowledge and modern scientific validation, positioning natural oral care formulations as effective alternatives or complements to conventional chemical-based products [143, 144].

## Traditional oral hygiene practices

The traditional oral hygiene practices of Indian ethnic minority communities reflect a deep understanding of local plants and their therapeutic value in maintaining oral health. There is extensive documentation showing that tribal knowledge systems in India utilize a wide range of medicinal plants to manage common oral conditions such as toothache, bleeding gums, halitosis, dental caries, and periodontal infections. The use of plant twigs and shoots as natural toothbrushes remains a core daily practice, with species such as *Azadirachta indica*, *Acacia nilotica*, *Emblica officinalis*, *Mimusops elengi*, and *Salvadora persica* serving both mechanical cleaning and antimicrobial functions. It is notable that powdered plant materials including *Curcuma longa*, *Cinnamomum tamala*, and *Achyranthes aspera* are traditionally applied as tooth-cleaning agents, reflecting an early form of herbal dentifrice. There is also widespread use of herbal mouth rinses prepared from *Ocimum sanctum*, *Mentha viridis*, *Psidium guajava*, and *Cocos nucifera*, which are valued for refreshing breath and reducing oral inflammation [145]. The management of toothache is particularly important in tribal healthcare, where healers associate pain with decay and inflammation and rely on analgesic plants such as *Piper nigrum*, *Phyllanthus emblica*, and *Syzygium aromaticum* for relief [146]. It is evident that bleeding gums and swelling are treated using anti-inflammatory and astringent plants like *Punica granatum*, *Allium sativum*, *Ficus benghalensis*, and *Acorus calamus*. There is recognition of halitosis as a disease marker, prompting the use of aromatic and cleansing herbs including *turmeric*, clove, and citrus species [145]. The integration of classical Ayurvedic practices such as gandoosha, kavalagra, and oil pulling using sesame oil highlights continuity between ancient medical texts and living traditions. It is important that sesame oil lignans such as sesamin and sesamolin contribute antioxidant and anti-inflammatory effects that support periodontal health [147]. The interest in natural products for oral health is particularly strong in regions with limited access to conventional dental care. Countries across Africa, Asia, and Latin America have long relied on traditional herbal remedies for maintaining oral hygiene. These include the use of chewing sticks made from plants such as *Diospyros mespiliformis*, *Diospyros lycioides*, and *Salvadora persica*, which possess intrinsic antimicrobial properties. While the ethnobotanical use of such products is well-documented, more research is needed to understand their clinical effectiveness and mechanisms of action [142].

## Challenges in translating natural products to clinical use and future prospective

Translating natural products and microbiome-based approaches into clinical practice is an exciting yet challenging path. One of the most fundamental issues is the limited ability to accurately analyze the microbiome. Current analytical methods still struggle to capture the full diversity, function, and metabolic activity of microbial communities. This gap restricts researchers from confidently identifying therapeutic candidates and assessing their safety and efficacy. It also slows the development of reliable microbiome biomarkers, which are essential for clinical studies and personalized treatments. Without validated methods, even diagnostic tools like microbiome-based IVD tests cannot be fully integrated into routine healthcare. A second challenge comes from the lack of consensus on what defines a “healthy” or “dysbiotic” microbiome. Because microbial communities vary widely among individuals, it is difficult to establish universal standards. This uncertainty complicates regulatory oversight and makes it harder to create clear guidelines for developing natural product-based therapeutics. As a result, companies, clinicians, and researchers may use different benchmarks, leading to inconsistent evaluations of product quality and performance.

Pharmacokinetic and pharmacodynamic assessments introduce further complexity. Natural compounds interact not only with host tissues but also with resident microbes that can metabolize or modify these compounds. Traditional models used to evaluate drugs do not fully capture these dynamic interactions. This means that results from preclinical studies often fail to predict clinical responses. Inter-individual variability in microbiome composition adds another layer of difficulty, as each person may react differently to the same natural product or microbial intervention. Safety concerns also remain a major barrier. Although natural products are often viewed as inherently safe, many possess strong bioactive properties. Potential toxicity, contamination, variability in plant sources, and interactions with medications all require careful examination. Microbiome-derived therapies, including donor-based products or oral microbiota transplantation, carry additional risks related to pathogen transfer, making rigorous screening essential. Personalized microbiome therapeutics offer exciting possibilities but bring regulatory and scientific challenges. Designing treatments based on an individual’s microbiome requires large datasets, advanced computational tools, and clear clinical frameworks that are still developing. Regulatory bodies currently do not have well-defined pathways for approving such personalized natural interventions, complicating their translation into practice. Effectiveness is another major concern. While many natural compounds show strong activity in lab studies, their performance in real-world human settings can be limited due to poor stability, low bioavailability, or rapid degradation in the oral cavity. Improving their clinical impact may require advanced delivery methods such as nanoencapsulation, slow-release dental coatings, or combinations with probiotics, prebiotics, or postbiotics.

## Conclusion

Translating natural products and microbiome-based strategies into clinical practice offers promising opportunities but remains fraught with scientific, technical, and regulatory challenges. It is clear that limitations in microbiome analysis, unclear definitions of healthy versus dysbiotic states, and highly variable microbial profiles continue to slow therapeutic development. Natural compounds, despite their strong laboratory performance, often face barriers related to stability, bioavailability, and safety, highlighting the need for robust clinical validation and advanced delivery systems. Microbiome-derived therapies also require strict screening to minimize risks, especially when donor-based products are involved. At the same time, the expanding understanding of oral microbiome–host interactions reinforce its importance in both oral and systemic disease prevention. Emerging tools such as nanotechnology, probiotics, postbiotics, and personalized interventions are steadily shaping a more precise therapeutic landscape. Future progress will depend on integrating multi-omics data, refining regulatory pathways, and ensuring that natural product-based therapies are effective, safe, and accessible for clinical use.

## Data Availability

This is a review paper. Data availability is not applicable.
